# Escalating doses of intravenous APAC demonstrate antithrombotic effect in pigs

**DOI:** 10.1186/s12959-025-00742-8

**Published:** 2025-06-04

**Authors:** Zsuzsa Bagoly, Annukka Jouppila, Rita Orbán-Kálmándi, Linda Lóczi, Dóra Bomberák, Zsófia Anna Kádár, Ádám Deák, Ádám Mátrai, Ildikó Beke Debreceni, János Kappelmayer, Norbert Németh, Riitta Lassila

**Affiliations:** 1 Division of Clinical Laboratory Science, Department of Laboratory Medicine, Faculty of Medicine, University of Debrecen, 98 Nagyerdei krt. Debrecen, Debrecen, H-4032 Hungary; 2 Lendület “Momentum” Hemostasis and Stroke Research Group of the Hungarian Academy of Sciences, Debrecen, Hungary; 3 Clinical Research Institute HUCH, Helsinki, Finland; 4 Research Program in Systems Oncology, Faculty of Medicine, University of Helsinki, Helsinki, Finland; 5 HUN-REN-DE Cerebrovascular Research Group, Debrecen, Hungary; 6 Department of Operative Techniques and Surgical Research, University of Debrecen, Debrecen, Hungary; 7 Department of Laboratory Medicine, Faculty of Medicine, University of Debrecen, Debrecen, Hungary; 8 Comprehensive Cancer Center, Department of Hematology, Unit of Coagulation Disorders, Helsinki University Hospital, Helsinki, Finland; 9 Aplagon OY, Helsinki, Finland

**Keywords:** Coagulation, Platelets, Pharmacodynamics, Drug development

## Abstract

**Background:**

Locally acting antiplatelet and anticoagulant (APAC) is developed as an antithrombotic agent for administration during vascular interventions and in thrombo-inflammatory conditions. APAC has entered human studies as a dual inhibitor of von Willebrand factor-mediated platelet recruitment on collagen and thrombin generation. We aimed to assess safety and escalating intravenous (i.v.) doses of APAC on hemostasis using a large animal model.

**Methods:**

We studied escalating APAC boluses (0.15–1.5 mg/kg; *n* = 11) and their reversal in anesthetized pigs for pharmacodynamics using functional coagulation testing. In some experiments, aspirin (500 mg) was co-administered with APAC, and protamine sulfate for reversal. Blood was repeatedly sampled for blood cell counts (CBC), activated partial thromboplastin time (APTT), prothrombin and thrombin time (PT, TT), thrombin generation (TG), activated clotting time (ACT), rotational thromboelastometry (ROTEM), and collagen-induced platelet aggregation (CIPA).

**Results:**

APAC was well-tolerated, and CBC remained stable. APAC modestly inhibited CIPA at high doses, while APTT, TT and ACT, unlike PT, prolonged dose-dependently. The anticoagulant ED_50_ doses of APAC and UFH showed similar range (0.54 vs. 0.43 mg/kg), but UFH lasted longer and was less reversible by protamine. At 0.75 mg/kg of APAC, TG was abolished, InTEM coagulation and clot formation times were prolonged *≥* 2.8-fold, maximum clot firmness was reduced to 8–45%, and amplitude to 35–80%. APAC effects were transient (T_1/2_ APAC = 30 min), and reversible by protamine.

**Conclusions:**

Escalating i.v. doses of APAC were safe and provided modest platelet inhibition.Our results indicate that the dose-dependent anticoagulation effects of APAC can be monitored using conventional laboratory assays.

**Supplementary Information:**

The online version contains supplementary material available at 10.1186/s12959-025-00742-8.

## Introduction

When vascular trauma occurs, platelets, as the first-in-line responders, adhere and aggregate at the injury site and confine coagulation activation to the site of vascular damage to cease the bleeding [1]. However, if uncontrolled, thrombus growth leads to vascular occlusion, ischemia, and organ failure. The standard of care for the prevention of arterial or venous thromboembolism includes systemic antiplatelet agents or anticoagulants [2, 3]. While the benefits of systemic antithrombotic therapy are indisputable during arterial thrombosis, the combined use of antiplatelets and anticoagulants needed for patient management increases their risk of bleeding [4]. The optimal target of the antithrombotic effect would be at the vascular site only, aligning with the natural physiology of the localized hemostasis.

Physiological hemostatic response to injury and pathological arterial thrombus growth both depend on von Willebrand factor (VWF), platelet activation upon collagen and other extravascular matrix proteins, and blood cell-mediated molecules [5, 6]. The adventitial layer of vasculature contains mast cells that can secrete heparin proteoglycans (Hep-PGs) upon stimulation [7]. Naturally occurring Hep-PGs are involved locally in the vasculature repair and in the regulation of coagulation. Secreted Hep-PGs also inhibit the growth of platelet thrombi on collagen under VWF-dependent blood flow conditions in vitro and reduce thrombotic process at the arterial anastomosis site in vivo in a small animal model [8–11]. The advantages of Hep-PGs have been utilized as the model for a bioconjugate having dual antiplatelet and anticoagulant (APAC) effects (Aplagon Ltd., Helsinki, Finland) [12].

In APAC, unfractionated heparin (UFH) chains are conjugated to albumin to form highly negatively charged glycosaminoglycan moieties [12]. APAC, similarly to Hep-PGs, but unlike isolated UFH chains, possesses unique simultaneous antiplatelet and anticoagulant properties.

APAC exerts its antithrombotic effect through multiple mechanisms. In a microfluidic flow model studying human blood under VWF-dependent arterial shear stress conditions, APAC as the sole anticoagulant (upon blood collection) reduced platelet and fibrin deposition on a surface coated with thrombogenic collagen and tissue factor (TF) in comparison with raw blood [13]. A similar local application effect was also observed in baboons [12]. As an intravenous anticoagulant, APAC prolonged clotting times, activated partial thromboplastin time (APTT) and thrombin time (TT) but not prothrombin time (PT) in vitro in human blood samples and in vivo in rodent and primate blood samples [12–15]. APAC was shown to bind to vascular injury exposed extracellular matrix to locally inhibit collagen- and thrombin-induced platelet aggregation, and to reduce platelet thrombus growth under VWF-dependent high blood shear rate conditions [13, 16, 17]. Furthermore, in irreversible ischemic reperfusion rat models, APAC prevented renal injury and inflammation, unlike UFH [18].

Due to its multifunctionality, APAC is being developed as an injury-targeting, locally acting antithrombotic agent for vascular interventions or treatment of arterial thrombosis and thrombo-inflammation. In this study, for the first time in a large animal model, we investigated the coagulation-related pharmacodynamic and pharmacokinetic effects of escalating, clinically relevant intravenous (i.v.) doses of APAC. The objective of this study was to assess clinical safety, escalating dose responses, co-administration with aspirin, half-life and reversibility with protamine sulfate, as tested by widely available conventional laboratory assays, including whole blood tests, which reflect both antiplatelet and anticoagulant effects.

## Materials and methods

### Materials

The following materials were used: APAC (heparin concentration 7.84 mg/ml, Aplagon Ltd., Helsinki, Finland & Cadila Pharmaceuticals Ltd, Ahmedabad, India), vehicle (137 mM NaCl, 10 mM Na_2_HPO_4_, pH 7.5), protamine sulfate (1400 anti-heparin IU/ml, Leo Pharma, Denmark), UFH (Heparin Leo, infusion solution, 5000 IU/ml, Leo Pharma, Denmark), and aspirin i.v. (500 mg, Bayer Vital, Leverkusen, Germany). UFH (IU/ml) dose was approximated to mg/kg by using the anti-Factor IIa activity of 180 UI/mg dry substance (according to the Ph. Eur. monograph 0333).

### Animal studies

Experiments were carried out according to the European Union Guidelines and Regulations on Animal Experiments with the approval of the University of Debrecen Committee of Animal Welfare (reg. Nr.: 3/2021/DEMAB). All methods reported are in accordance with ARRIVE guidelines. All procedures were performed in 3-month-old female Hypor pigs (*n* = 13, weight 25.6±1.5 kg) under general anesthesia. For premedication, 2 mg/kg azaperone (Elanco GmbH, Cuxhaven, Germany) was used (intramuscularly) i.m., together with 0.03 mg/kg atropine (Egis Pharmaceuticals PLC, Budapest, Hungary). Anesthesia was induced with 2 mg/kg xylazine and 20 mg/kg ketamine i.m. (Produlab Pharma BV, Raamsdonksvee, The Netherlands), and for maintaining narcosis 1 mg/kg xylazine and 10 mg/kg ketamine were used. Animals were intubated with a PVC endotracheal tube (Eickemeyer, Tuttlingen, Germany) for assisted ventilation. The left external jugular vein was cannulated to administer medications, while the right external jugular vein was used for blood sampling. Animals were under general anesthesia throughout the study and were euthanized by anesthetic overdose at the end of the experiment. During the method of euthanasia, the animal was deeply sedated, and a high dose of pentobarbital (100 mg/kg) was administered to cause a painless death. The use of euthanasia was justified in the study protocol and approved by the ethical review board. Anesthesia and euthanasia were performed by trained personnel and the above-described methods were chosen as the most appropriate for the study and the animals’ welfare.

During blood collection, the first 0.5 ml of blood was discarded, followed by sampling directly to standard tubes (Vacuette, EDTA K2 or 3.2% sodium citrate blood collection tubes, Greiner Bio-One, Kremsmünster, Austria). At the end of each blood sampling, the catheter was flushed with 2 ml of saline (0.9% NaCl). Vehicle, escalating doses of APAC or UFH (0.25 mg/kg to 1.5 mg/kg each) or 500 mg aspirin were administered (1 ml volume) as an i.v. bolus.

### Design and execution of the study

**Fig. 1:**
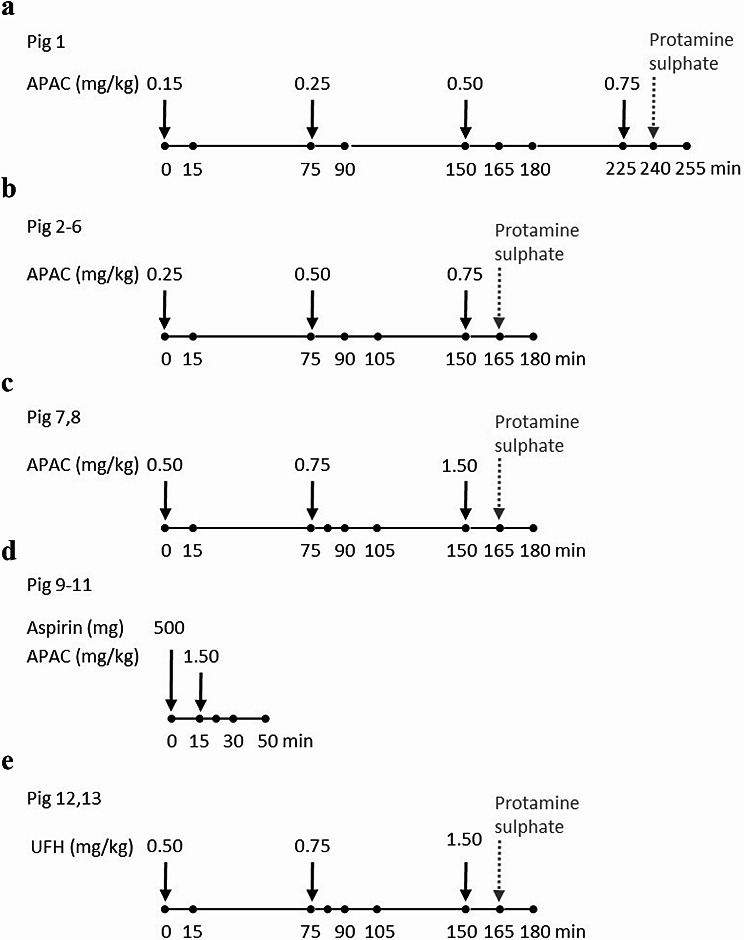
Design and execution of the study. Time points for administration of APAC or UFH (black arrows) and timing of blood sample collections (black points) for subsequent analysis of pharmacodynamic variables are shown. At timepoints indicating simultaneous administration of APAC or UFH and blood collection, blood was collected before the dosing. Escalating doses of APAC were assessed in Pig 1 (*n* = 1) to select the initial dose levels (**a**), in Pig 2–6 (*n* = 5) for comparative repeated dosing (**b**), and in Pig 7 and 8 (*n* = 2) to estimate the effects at a supra-high dose (**c**). Schedule for the co-administration of aspirin and APAC (*n* = 3) is shown in Panel **d**. UFH was administered in Pig 12 and 13 (*n* = 2) (**e**). Protamine sulfate (140 IU/kg) was studied as a reversal agent for APAC or UFH at the end of the experiments

The overall study flow is presented in Fig. 1. Baseline blood sample was collected before administering the first APAC dose, followed by collection times for the specified dose-regimes. Experimental set-up logistics, pre-analytics and the method of blood sampling were tested in the first animal (Pig 1). Overall, our logistic was to reduce the number of animals to be studied and used stepwise dose escalation to cover a relevant range and various methods of blood testing. In the next animals, escalating doses of APAC were administered at various time points (0, 75 and 150 min), ranging from low doses (0.25, 0.5 and 0.75 mg/kg, [Pigs 2–6]) to high doses (0.5, 0.75 and 1.5 mg/kg, [Pigs 7,8]). Multiple dosing regimens were used to keep experiments within reasonable timeframe (<3 h), to avoid physiological effects of prolonged anesthesia.

Protamine sulfate (140 IU/kg) was administered in Pigs 1–8, at 15 min after the highest dose of APAC (Pig 1 at 240 min, and Pigs 2–8 at 165 min) to assess anticoagulation reversal. The co-administration of aspirin (500 mg) and APAC (1.5 mg/kg) was studied in three animals (Pigs 9–11). Baseline blood was collected before aspirin was administered. In case of two animals, UFH was used instead of APAC at escalating doses (0.5, 0.75 and 1.5 mg/kg, [Pigs 12–13]).

### Laboratory investigations and specific measurements of hemostasis

All blood samples were transported to the laboratory immediately and measured within 1 h after the collection. Complete blood cell count (CBC), using EDTA-anticoagulated blood was studied with an ADVIA-2120i automated hematology analyser (Siemens, Forcheim, Germany), using a porcine-specific cell count software. For coagulation assays, plasma was separated from citrated blood by double centrifugation (1500 x g, twice at 15 min, room temperature) and the tests were performed immediately after plasma separation using a BCS-XP coagulometer (Siemens Healthcare Diagnostics, Marburg, Germany). PT, APTT and TT were assessed using a human recombinant thromboplastin reagent (Dade Innovin), standard APTT reagent (Actin FS), and bovine thrombin reagent (Siemens Healthcare Diagnostics Products, GmbH, Marburg, Germany) respectively, according to the manufacturer’s instructions. Fibrinogen levels were measured with a BCS-XP coagulometer by the standard method of Clauss (Siemens Healthcare Diagnostics Products, GmbH, Marburg, Germany). For the execution of the TG test, aliquots of double centrifuged citrated plasma were stored at -80 °C until analysis.

Rotational thromboelastometry (ROTEM; InTEM, ExTEM, FibTEM and ApTEM) was performed in citrate-anticoagulated blood within 1 h after blood sampling, using the ROTEM Sigma device and the Sigma Complete Cartridge kit (Werfen, Barcelona, Spain). The following ROTEM variables were recorded for all: CT (coagulation time), CFT (clot formation time), MCF (maximum clot firmness), A5-A30 (clot firmness/amplitude measured at various times between 5 and 30 min) and LI30-LI60 (lysis index measured at various times between 30 and 60 min).

Activated clotting time (ACT) was measured according to the manufacturer’s instructions immediately after blood collection using a point-of-care device (Hemochrom Jr., Accriva Diagnostics Inc., San Diego, CA, USA).

The TG test was conducted with previously established protocols, utilizing the Thrombinoscope CAT assay (Calibrated Automated Thrombogram, Maastricht, The Netherlands), according to the manufacturer’s guidelines (Diagnostica Stago, Asnières, France) using PPP-Reagent™, containing 5 pM recombinant TF and 4 µM phospholipids. For details, please refer to references [19, 20]. All samples were processed in duplicate. Fluorescence signals were detected using a Fluoroskan Ascent^®^ fluorimeter (Thermo Fisher Scientific, Waltham, MA USA) and the resulting curves were analyzed using the Thrombinoscope software (Thrombinoscope BV Maastricht, The Netherlands). The TG parameters were calculated and presented by the Thrombinoscope software: 1/ Lagtime, defined as the signal deviation point exceeding 2 standard deviations (SD) from the baseline; 2/ Endogenous Thrombin Potential (ETP), the area under the curve; 3/ Peak Thrombin (Peak), the highest thrombin concentration reached; 3/ Time to Peak (ttPeak), the duration to achieve peak TG [20].

Colagen-induced platelet aggregation (CIPA) using 1 µg/ml fibrillar collagen as an agonist (Hart Biologicals, Hartlepool, UK) was measured in PRP (120 g, 15 min, room temperature) with Chrono-Log model 700 lumi-aggregometer (Chrono-Log Corporation, Havertown, PA, USA) within 1 h of blood collection. Platelet counts of PRP were adjusted to 300 × 10^9^/l by adding platelet-poor plasma (PPP, twice 1500 x g, 15 min, room temperature). Results were expressed as the extent of maximal aggregation (∆ transmission %).

### Statistical analysis

Data were analyzed with GraphPad Prism (GraphPad Software, La Jolla, CA, USA). The Shapiro-Wilk test was used to test for normality. Continuous variables were expressed as mean ± SD. For paired data, Student’s t-test or two-way ANOVA was applied to compare differences between groups. A p-value < 0.05 was considered statistically significant.

## Results

### APAC effects on coagulation screening tests

All treated animals (Pigs 1–8) tolerated APAC well without adverse reactions or visible signs of bleeding during any experiment. Blood cell counts remained stable with less than 10% variation up to the last APAC administration, except for Pig 4, in which leukocyte count decreased by 20%. After administration of protamine sulfate to APAC-treated animals platelet counts were reduced on average by 11 ± 6%, (range 4–22%) (Supplemental Figure S1).

**Fig. 2:**
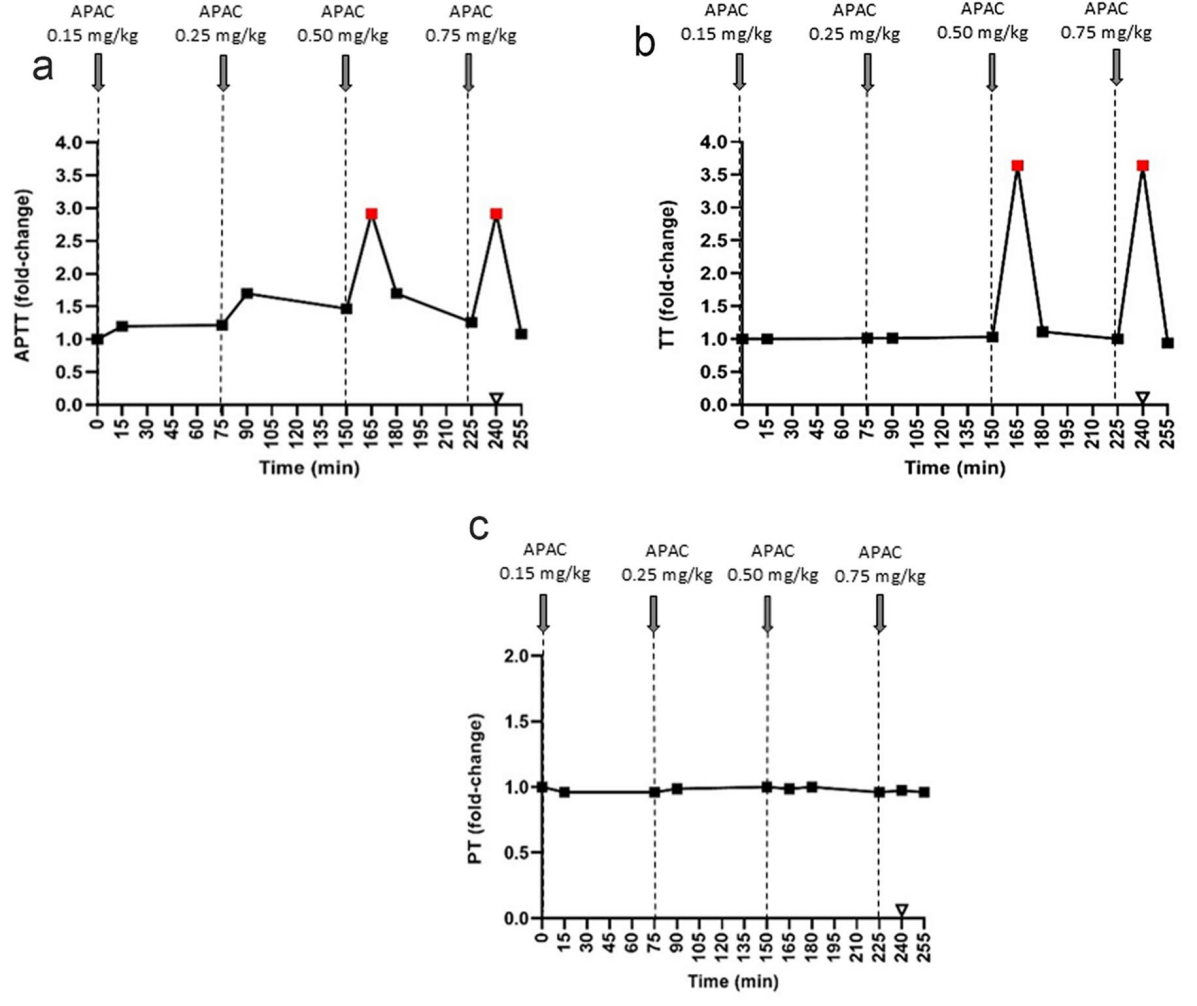
Assessment of effective doses of intravenous APAC by activated partial thromboplastin time (APTT), thrombin time (TT) and prothrombin time (PT) in plasma. APTT (**a**), TT (**b**) and PT (**c**) were followed in plasma after administering escalating doses of APAC (0.15, 0.25, 0.5 and 0.75 mg/kg) (Pig 1; *n* = 1). The time points of APAC dosing were 0, 75, 150, and 225 min. Blood collection times and respective results are presented by solid symbols. Results represent relative to the baseline value (APTT, 68 s; TT, 28 s and PT, 7.7 s). Protamine sulfate (140 IU/kg, open triangle) was administered as an antidote to all animals at 15 min after the highest APAC dose (at 240 min). Maximal APTT and TT detection times were 200 s and 100 s, respectively. Red symbols indicate the reach of the maximal detection time (upper limit of detection)

First, one pig was used to investigate the anticoagulant effect of APAC with coagulation screening tests (Fig. 2). APAC dose-dependently prolonged both APTT and TT. APAC (0.15 mg/kg) prolonged APTT by 20% after 15 min, while the other clotting times remained at the baseline level. At 0.25 mg/kg APAC prolonged APTT 1.7-fold versus the baseline, while the upper limit of detection (200 s) was reached at 0.5 mg/kg. TT was affected by APAC doses above 0.5 mg/kg and reached the upper limit of detection (100 s) at higher doses.

**Fig. 3:**
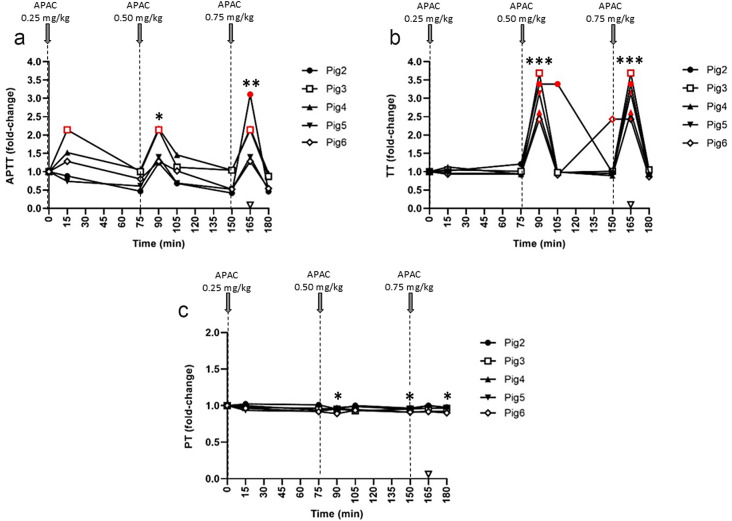
Effect of escalating doses of intravenous APAC on activated partial thromboplastin time (APTT), thrombin time (TT) and prothrombin time (PT) in plasma. The results represent fold-changes from the baseline on APTT (**a**), TT (**b**) and PT (**c**) followed in plasma after administering escalating doses of APAC at 0.25, 0.5 and 0.75 mg/kg (Pig 2–6; *n* = 5). The time points of APAC dosing were at 0, 75, and 150 min. Blood collection times are presented by solid symbols. Results are presented in relation to baseline values (ranges for APTT 35–156 s, for TT 27–41 s and for PT 8–11 s). Protamine sulfate (140 IU/kg, open triangle) was administered as an antidote to all animals at 15 min after the highest APAC dose (at 165 min). Maximal APTT and TT detection times were 200 s and 100 s, respectively. Red symbols indicate the reach of the maximal detection time. Please refer to Supplemental Figure S2 for individual data. *p < 0.05, **p < 0.01, ***p < 0.001, relative to the baseline value

In Pigs 2–6, APTT was prolonged in 3/5 animals at the lowest APAC dose (0.25 mg/kg), while higher doses prolonged APTT in all animals (Fig. 3a). Notably, the effect of APAC on APTT was reversible, returning to baseline in all pigs before administrating the next dose; moreover, protamine sulfate effectively reversed the effect of APAC (0.75 mg/kg) (Fig. 3a). The results of TT were similar, although this screening test was less sensitive than APTT, not prolonged by the lowest APAC dosing (Fig. 2b). PT was unaffected by all APAC doses (Figs. 2c and 3c), and fibrinogen levels were also unaltered during the experiment (data not shown).

### APAC effects on ROTEM

**Fig. 4:**
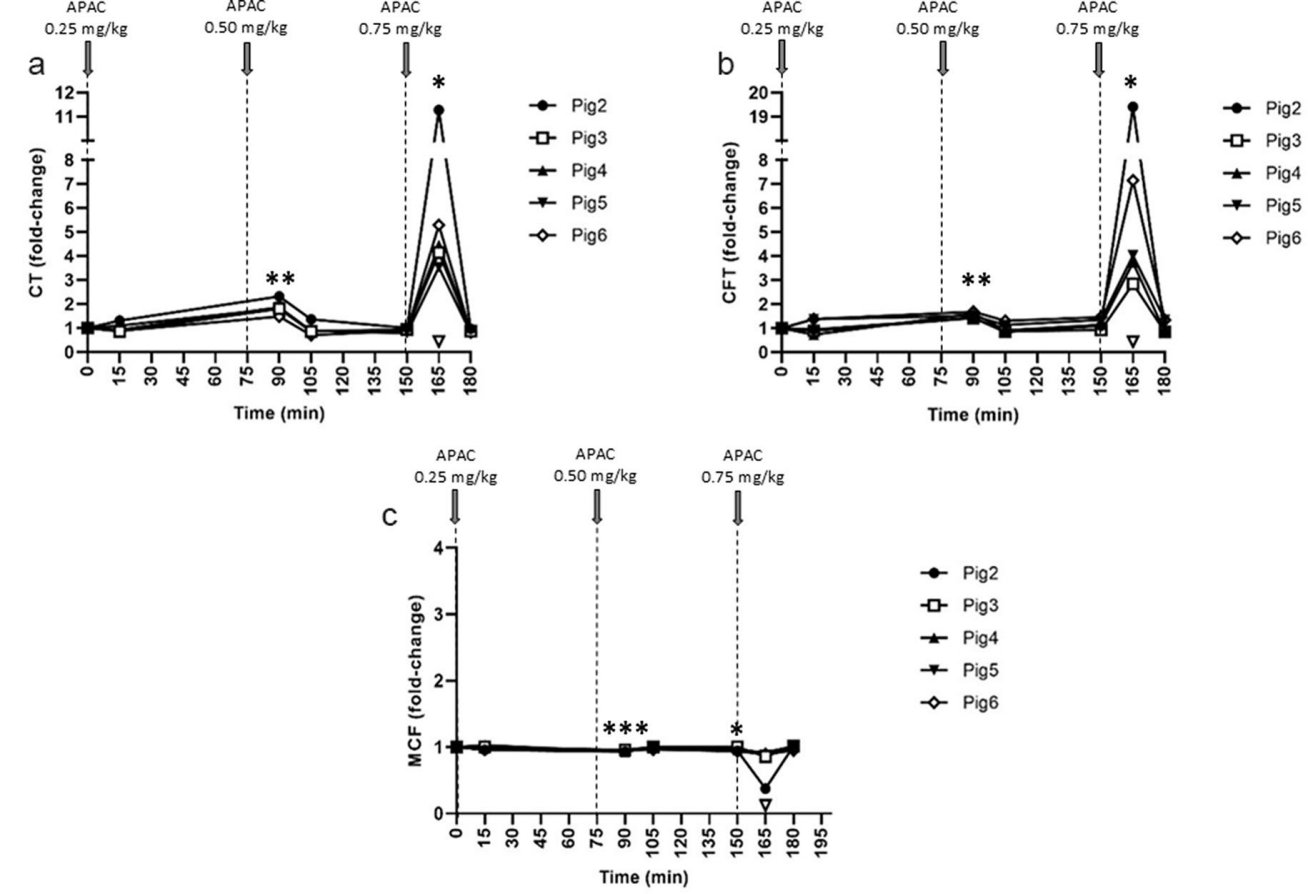
Effect of escalating doses of intravenous APAC on intrinsic pathway-triggered blood coagulation InTEM. Administration of escalating doses of APAC was followed with rotational thromboelastometry (ROTEM; InTEM) at 0.25, 0.5 and 0.75 mg/kg (Pig 2–6; *n* = 5). Variables reflecting the rate of initial fibrin formation (coagulation time, CT) (**a**), the interaction of platelets and fibrin to form a stable clot (clot formation time, CFT) (**b**), and maximum clot firmness (MCF) (**c**) are shown. The time points of APAC dosing were at 0, 75, and 150 min. Blood collection times are presented by solid symbols. Results are presented in relation to baseline values (ranges for CT 90–145 s, for CFT 26–47 s and for MCF 71–82 s). Protamine sulfate (140 IU/kg, open triangle) was administered to all animals at 15 min after the highest APAC dose (at 165 min), and it uniformly reversed the effect of APAC. *p < 0.05, **p < 0.01, ***p < 0.001, relative to the baseline value

Increasing doses of APAC (0.25, 0.5 and 0.75 mg/kg) were also followed by ROTEM (Fig. 4). The effect of APAC was detectable by the CT, CFT and MCF parameters of the InTEM channel (Fig. 4a-c), while the parameters of ExTEM, FibTEM and ApTEM channels remained unchanged during each dose of APAC. The anticoagulant effect of APAC on InTEM clot formation was most pronounced at the highest dose, but one animal (Pig 2) showed an exceptionally strong anticoagulation. The longitudinal effect of APAC as detected by ROTEM was reversible, and protamine sulfate fully reversed anticoagulation at the end of the experiment.

### APAC effects on thrombin generation in calibrated automated thrombogram

TG was determined from plasma by the CAT at baseline and after dose escalations of APAC (0.25, 0.5 and 0.75 mg/kg, Pig 2–6) at 15, 30, or 75 min (Supplemental Table S1). After 15 min of APAC at 0.25 mg/kg, TG was unaffected, whereas at the 0.5 mg/kg dose, TG was abolished (in 2/5 pigs) or reduced (in 3/5 pigs). In all pigs, during the follow-up, TG normalized (Supplemental Table S1).

At the 0.75 mg/kg dose, TG was uniformly abolished at 15 min but reverted to baseline after administration of protamine sulfate.

### Comparison of APAC effects with UFH

**Fig. 5:**
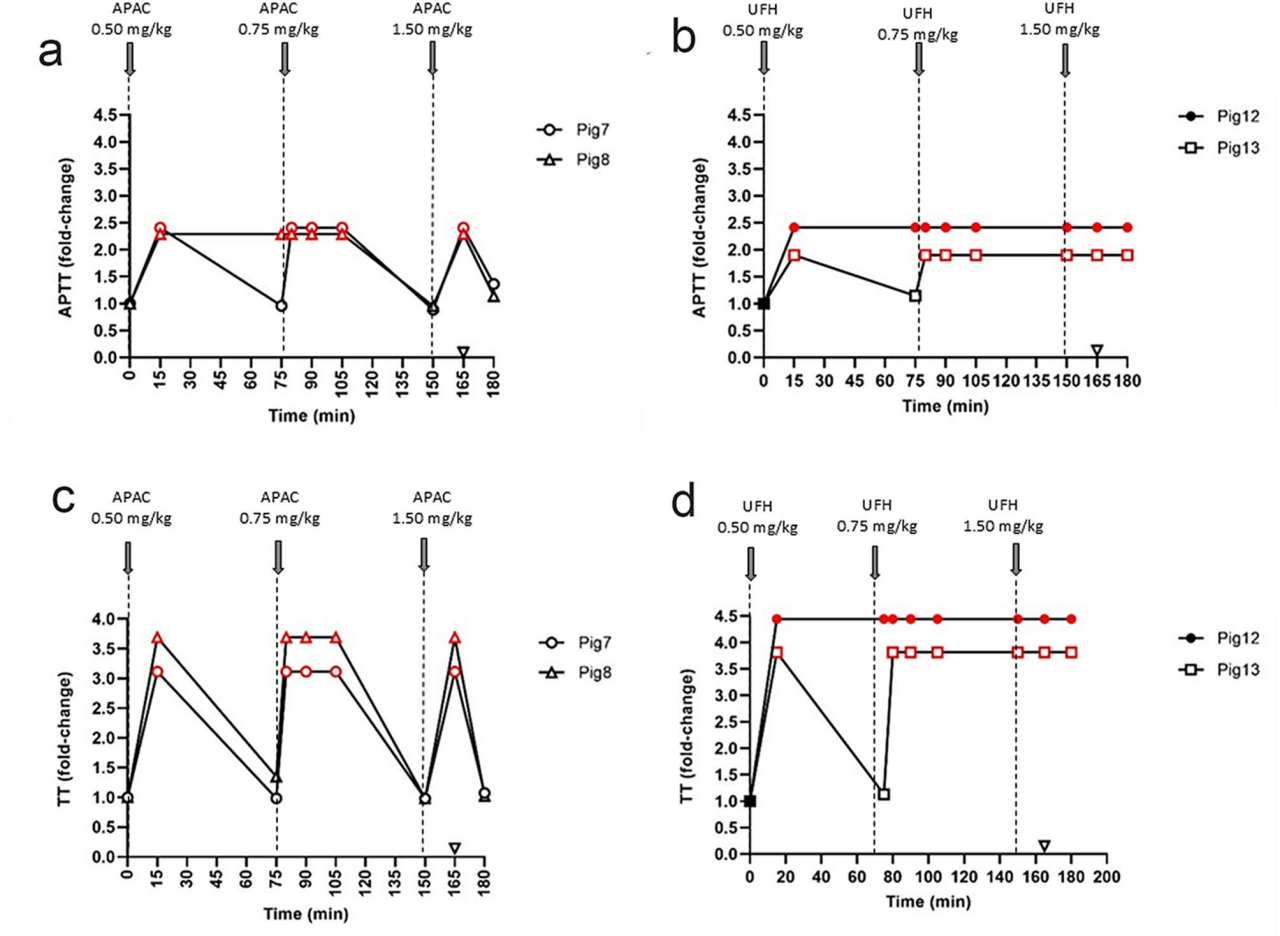
Effect of escalating doses of intravenous APAC or UFH on activated partial thromboplastin time (APTT) and thrombin time (TT) in plasma. APTT and TT were followed in plasma after the escalating dosing of APAC (0.5, 0.75 or 1.5 mg/kg) in Pigs 7–8 (*n* = 2) (**a** and **c**). The dosing time points for APAC or UFH were 0, 75, and 150 min. The effect of UFH was tested at 0.5, 0.75 and 1.5 mg/kg similarly in Pigs 12–13 (*n* = 2) (**b** and **d**). Blood collection time points are presented by solid symbols. Results are shown in relation to baseline values (ranges for APTT 83–105 s, and for TT 22–32 s). Protamine sulfate (140 IU/kg, open triangle) was administered to all animals 15 min after the highest APAC or UFH dose (165 min). The maximal detection time for APTT was 200s, and for TT 100 s. The red symbols indicate the reach of maximal detection time

**Fig. 6:**
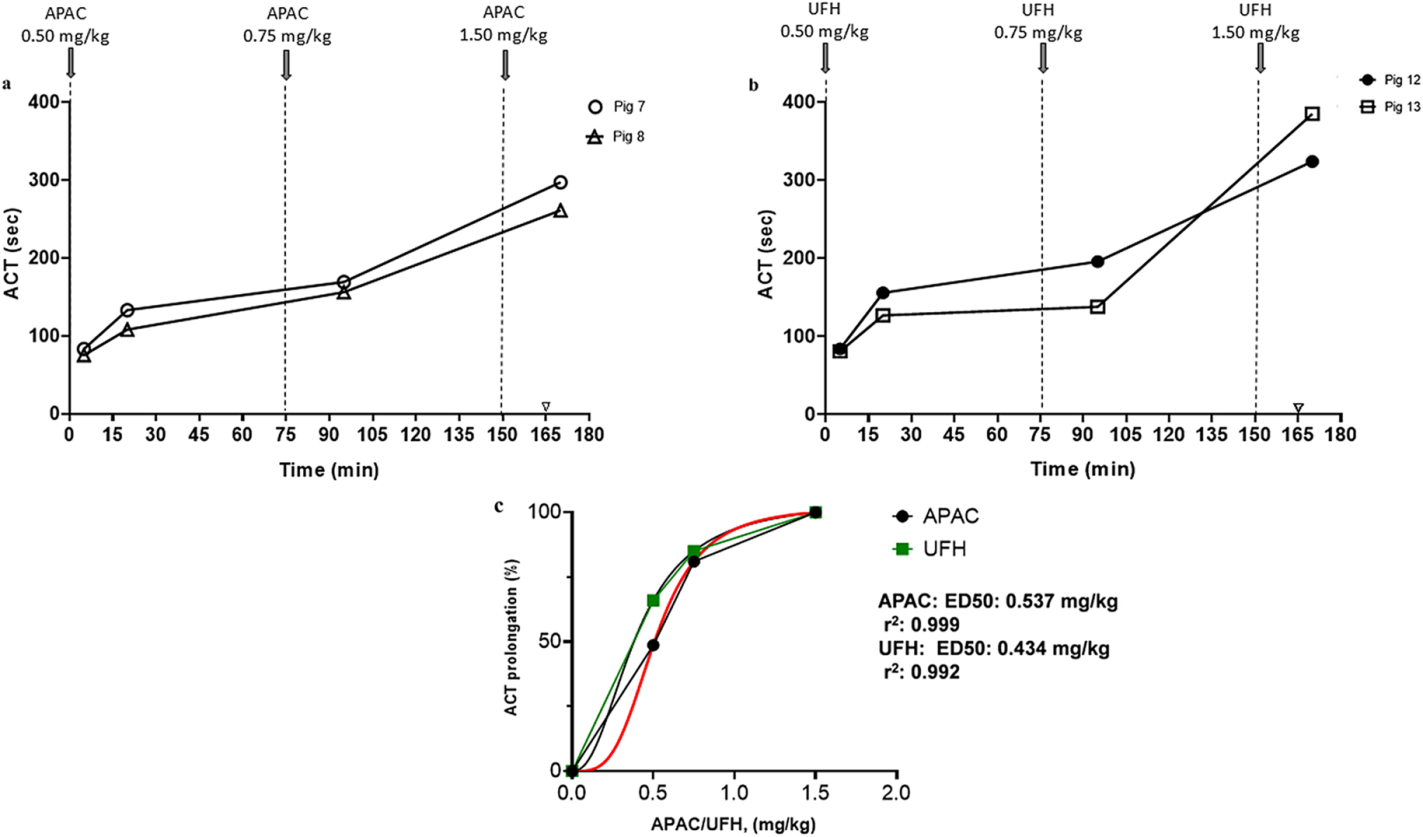
Effects of escalating intravenous APAC or UFH doses prolonged activated clotting time (ACT) in whole blood. ACT after administration of escalating doses of APAC at (0.5, 0.75 or 1.5 mg/kg) (panel **a**, Pig 7–8; *n* = 2) or UFH at 0.5, 0.75 and 1.5 mg/kg (panel **b**, Pigs 12–13; *n* = 2). Prolongation of ACT as compared to baseline (%) vs. the dose of anticoagulant is shown on panel **c** (dose-response curves, APAC: red, UFH: green). ED_50_: effective dose needed for 50% of the effect

The anticoagulant effects of APAC were compared to that of UFH using the high escalating doses of 0.5, 0.75 and 1.5 mg/kg (Figs. 5 and 6). As expected, based on the results obtained with the lower doses, higher doses of APAC (0.5–1.5 mg/kg) resulted in APTT prolongation. The effect spontaneously reversed at 0.75 mg/kg of APAC (Fig. 5a), unlike for UFH (Fig. 5b). Again, reversible effects of APAC and irreversible effects of UFH were observed in TT at the 0.75 mg/kg dose level (Fig. 5c and d). PT was modestly prolonged at the highest dose in case of both drugs (data not shown).

Administration of protamine sulfate (140 IU/kg) reversed the effect of APAC, as detected by APTT and TT (Fig. 5a and c), whereas it was ineffective at the same dose of UFH (Fig. 5b and d). Based on our calculations, the half-life of APAC is 35 min in pigs, while that of UFH is two-fold longer, T_1/2_ =90 min, as previously reported [21].

The anticoagulation was most visible by ACT monitoring after escalating doses of APAC or UFH at 0.5–1.5 mg/kg (Pig 7–8 and Pig 12–13, respectively) (Fig. 6). Based on the prolongation of ACT, the calculated 50% effective doses (ED_50_) of APAC and UFH were quite similar (0.54 mg/kg vs. 0.43 mg/kg).

### Effect of APAC on collagen-induced platelet aggregation

**Fig. 7:**
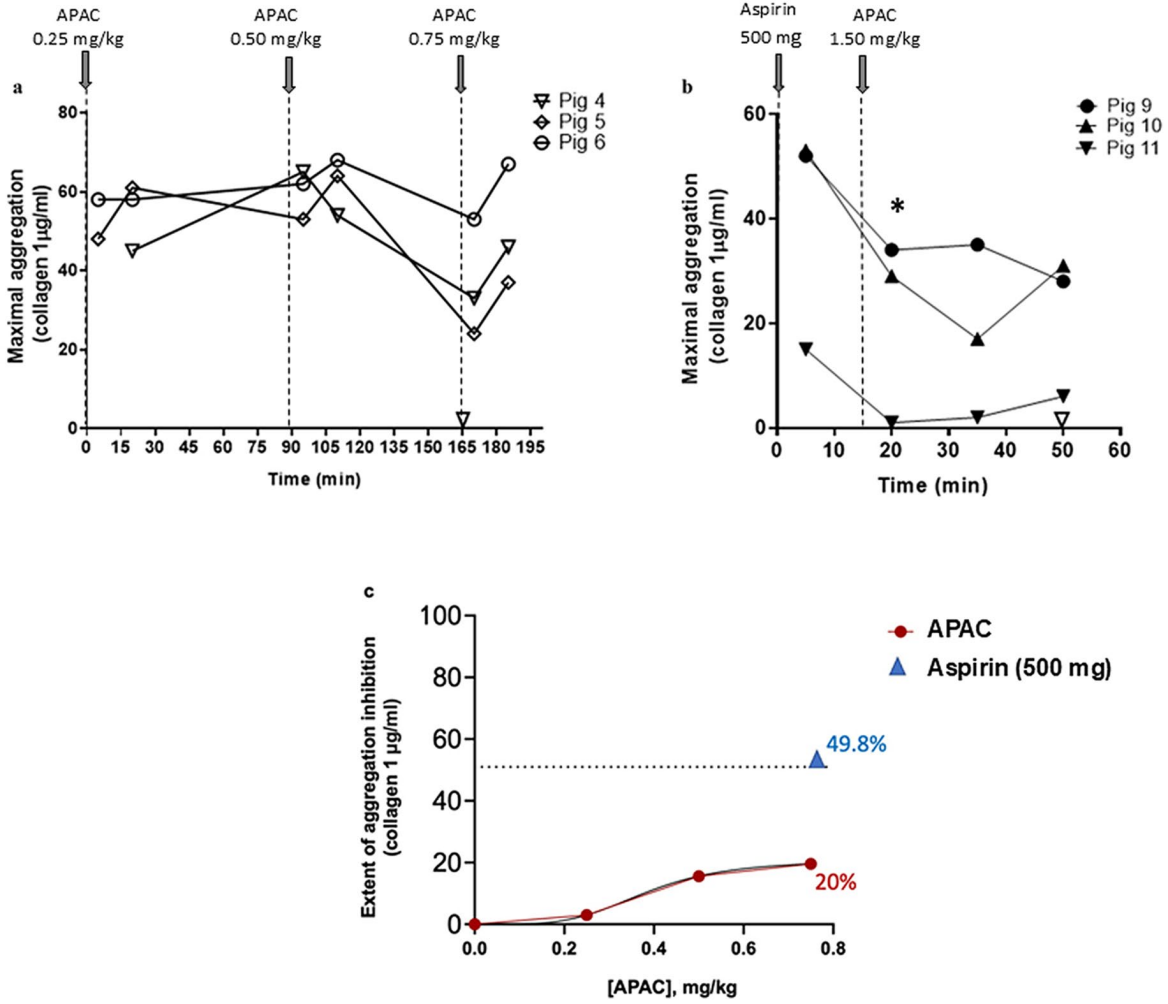
Antiplatelet effects of APAC alone and co-administered with aspirin. Collagen-induced platelet aggregation (CIPA) at increasing administered doses of APAC (0.25, 0.50 and 0.75 mg/kg) (Panel **a**, Pig 4–6; *n* = 3). Co-administration of aspirin (500 mg) and APAC (1.5 mg/kg) were carried-out in three animals and followed by CIPA (Panel **b**, Pigs 9–11; *n* = 3). Results are expressed as the extent of maximal aggregation (∆transmission %). The dose-response effect of APAC on CIPA (red circles and solid lines) as compared to 500 mg aspirin alone (blue triangle and dotted line) (**c**). *p < 0.05, relative to the baseline value

To monitor the antiplatelet effect of APAC, CIPA was carried out at increasing doses of APAC (0.25, 0.50 and 0.75 mg/kg) administered to Pigs 4–6 (Fig. 7). Decreased platelet aggregation was observed only at the highest dose studied (0.75 mg/kg), and it showed a reversible pattern of inhibition, similarly to the anticoagulant profile (Fig. 7a). The antiplatelet effect of APAC at this dose was limited, more than two-fold weaker than the effect of 500 mg aspirin as measured by CIPA. Of note, when APAC was co-administered with 500 mg aspirin, the high-dose APAC of 1.5 mg/kg did not further inhibit (in 2/3 animals) platelet aggregation as compared to aspirin alone (Fig. 7b). Overall, the inhibitory effect of APAC on CIPA statistically significant, but modest, being 20% at the highest APAC dose administered, as compared with 50% inhibitory effect of 500 mg aspirin (Fig. 7c).

## Discussion

This study presents novel findings on the i.v. administration of escalating APAC dosing in anesthetized pigs, highlighting its safety, dose-dependent anticoagulant effects, transient action, and reversibility with protamine sulfate. Our results indicate that APAC doses can be easily monitored using standard laboratory assays, making it a promising candidate for localized antithrombotic therapy during vascular interventions. We also demonstrated for the first time using i.v. dosing of APAC in a large animal model that clinically relevant coagulation tests, including whole blood ACT and ROTEM, can depict the dose escalation. Compared with UFH, APAC demonstrated a shorter half-life and more controlled hemostatic effects, which may reduce bleeding risk. APAC, unlike UFH temporarily inhibited CIPA. Also, APAC dose-dependently, but transiently prolonged coagulation times of APTT, and TT, while PT was not affected, alike UFH. According to ACT, APAC showed anticoagulant efficacy with an ED_50_ of 0.54 mg/kg. APAC was most sensitive when measured by TT, which prolonged exponentially from baseline of 0.25 mg/kg to the limit of detection at the doubled dose (0.5 mg/kg), an observation that we have also noted in first-in-human Phase I safety studies as well (unpublished).

During the experiment, no signs of hemorrhagic complications were observed, and in general CBC were unaffected, supporting hemostatic safety of APAC. Pigs had differences in their baseline CBC values (Supplemental Figure S1), and the difference in leukocyte levels was 2.7-fold, which may have caused some variability between animals. At the last time point, after the protamine administration, platelet count decreased by 4–22% (APAC 0.15 to 1.5 mg/kg) from the baseline (Supplemental Figure S1). Of note, the co-administration of aspirin in vivo in this pig model did not trigger bleeding or enhance the inhibitory effect of APAC on CIPA, referring to safety when co-administering aspirin.

APAC did not affect fibrinogen levels at any of the applied doses. In whole blood ROTEM analysis, APAC prolonged intrinsic pathway triggered coagulation (CT) already at the lowest dose (0.25 mg/kg), while the dual antiplatelet and anticoagulant effects (CFT and MCF) were effective at the higher dose level of *≥* 0.75 mg/kg only. This can be approximated as the concentration of 10 µg/ml APAC (as the sole anticoagulant), used in human blood perfusion experiments [13]. In our previous study, the dual inhibition of platelet (50%) and fibrin (> 90%) recruitment on a surface covered with both collagen and TF occurred by APAC under the high shear rate of 1000 1/s, unlike at the low shear of 200 1/s, where only the anticoagulant effect of APAC could be observed [13].

The porcine model was chosen as the large animal model for the functional pharmacodynamic studies due to the availability of sufficient blood sample needed for comprehensive analyses. The dose escalation allowed us to detect the antiplatelet and anticoagulant dose responses in healthy animals. However, interspecies differences in hemostasis must be considered as the hemostatic system of pigs has been reported to be somewhat more thrombogenic than that of humans or other species [22–24]. Notably, pigs have nearly twice the platelet count of humans, which may contribute to this heightened thrombogenicity. Compatibly, APAC’s antiplatelet potency in CIPA was less evident in porcine PRP, since the 0.75 mg/kg dose was needed to reduce maximal platelet aggregation, in comparison to human PRP where similar results by in vitro spiking would be achieved with only 70–90% of that dose [14, 16]. Similarly, in mouse platelets, APAC (> 2.5 µg/ml) very effectively inhibited the aggregation of in vitro-spiked PRP [12, 14, 15, 16, 17]. A review by *Staelens et al.* highlighted that elevated platelet counts in pigs, relative to humans, may enhance thrombotic potential [24]. In pigs the intrinsic pathway triggered coagulation in ROTEM by CT and CFT resembles that of human blood, however, MCF reflecting platelet involvement is significantly greater [23]. Notably, pigs are often used as animal models in research on hemostasis, and it has been reported that human functional coagulation assays are useful in porcine plasma [25]. As the clinical application of APAC will initially target the prevention of intervention-related or the treatment of arterial thrombotic events, the above-described pig model, despite some inevitable limitations, seemed optimal to test the antiplatelet and anticoagulant effects of APAC by conventional functional hemostasis laboratory methods, before further exploration in clinical studies.

The antiplatelet activity of APAC (0.3 and 0.5 mg/kg) has been evident in vivo in two different carotid thrombosis models in mice, induced either by exposing collagen-rich matrix on arterial wall or by laser-induced carotid artery damage [16]. In two non-human primate models APAC has inhibited both platelet deposition and fibrin formation by 50% in collagen-coated graft model ex vivo, and inhibited arterial occlusion when applied at the site of stenosis in crush-injured femoral artery in a modified Folts model [12]. These above supportive data have paved the way for the ongoing clinical studies of APAC using (1) local administration upon creation of arteriovenous fistula to assess its maturation and patency, and (2) systemic (i.v.) application to assess safety of the transient antiplatelet and anticoagulant effects.

Here, the repeated and escalating bolus doses of APAC lead to a short-lasting systemic exposure, in line with the therapeutic aim of a localized action and reduction of the bleeding risk via less generalized platelet and coagulation effects [2, 26]. The pharmacodynamics of the present observations are also in accordance with long-term pharmacokinetic profile of APAC in the toxicology studies [15]. There, after the 14-day repeated daily dosing in rodents and primates, according to APTT and TT, APAC (10 and 6 mg/kg, respectively) responses reverted to baseline levels within 1.5 (< 10 mg/kg) or essentially by 6 h [15]. Protamine sulfate reversed APAC according to all coagulation tests, and baseline values were reached even after the dose escalations. For reversal of anticoagulant therapy, these data are supportive, should a high i.v. APAC dose be accidentally administered [27].

We used coagulation tests as the surrogate pharmacokinetic markers of APAC and UFH, in the absence of a direct method to quantify APAC in plasma. Recently published studies reported new possibilities of monitoring heparin concentrations in plasma by utilizing liquid chromatography-mass spectrometry and multiple reaction monitoring, which we are currently addressing [28]. To pave the way for clinical applications of APAC, the availability of routine laboratory monitoring with the current data supports that these methods can be easily applied in patients. Our results show that APAC provides controllable and reversible antithrombotic effects, supporting APAC as a promising candidate for further clinical investigation in settings requiring such properties.

## Conclusions

We aimed to evaluate the clinical safety and dose-dependent measurability of APAC compared to UFH in a large animal model using pigs. Although pigs differ from humans—exhibiting stronger coagulation function, higher thrombin activity, and elevated platelet counts—escalating doses of APAC led to a dose-dependent prolongation of coagulation times and inhibition of both the intrinsic pathway and thrombin-triggered coagulation in vivo, with modest antiplatelet effects.

All hemostasis effects observed under the ascending dose regimen of APAC were transient and less pronounced than those induced by UFH. Unlike APAC, UFH lacks antiplatelet properties. Notably, APAC could be effectively monitored using widely available and established coagulation assays as well as point-of-care whole blood methods.

These findings suggest that APAC may serve as a convenient dual antithrombotic agent during vascular interventions, potentially reducing the hemostatic risks associated with the coadministration of separate antithrombotics during arterial procedures. This approach could help address limitations of traditional systemic treatments. Further research and clinical trials are warranted to determine its efficacy and broader applicability in the prevention and treatment of thrombotic conditions.

## Electronic supplementary material

Below is the link to the electronic supplementary material.

Supplemental Figure S1 (PDF): White blood cell (WBC), red blood cell (RBC), hemoglobin (HGB) and platelet count (PLT) during experiment time in pigs 1-8.

Supplemental Table S1 (PDF): Effect of escalating doses of intravenous APAC on thrombin generation in plasma and its reversal by protamine sulfate in pigs 2-6.

Supplemental Figure S2 (PDF): Effect of escalating doses of intravenous APAC on activated partial thromboplastin time (APTT), thrombin time (TT) and prothrombin time (PT) in plasma.

## Data Availability

The data that support the findings of this study are not openly available due to reasons of sensitivity and are available from the corresponding author upon reasonable request.

## Abbreviations

ACT: Activated clotting time
ADP: Adenosine-diphosphate
A5-A30: Clot firmness/amplitude at 5–30 min
APAC: AntiPlatelet AntiCoagulant
APTT: Activated partial thromboplastin time
CBC: Complete blood cell count
CFT: Clot formation time
CIPA: Collagen-induced platelet aggregation
CT: Clotting time
ED50: 50% effective dose
ETP: Endogenous thrombin potential
Hep-PG: Heparin-proteoglycan
i.v.: Intravenous
LI30-LI60: Lysis index measured at various times between 30 and 60 min
MCF: Maximum clot firmness
min: Minute
PPP: Platelet poor plasma
PRP: Platelet-rich plasma
PT: Prothrombin time
PVC: Polyvinyl chloride
ROTEM: Rotational thromboelastometry
SD: Standard deviation
TF: Tissue factor
TG: Thrombin generation
TT: Thrombin time
ttPeak: Time to peak
UFH: Unfractionated heparin
VWF: Von Willebrand factor
