# Supplementary material for: Escalating doses of intravenous APAC demonstrate antithrombotic effect in pigs
Source: Thromb J. 2025 Jun 4;23:57. doi: 10.1186/s12959-025-00742-8 (PMC12135276; doi:10.1186/s12959-025-00742-8)
Supplement: Supplementary file 1 — Supplemental Figure S1 (PDF): White blood cell (WBC), red blood cell (RBC), hemoglobin (HGB) and platelet count (PLT) during experiment time in pigs 1-8. [file 12959_2025_742_MOESM1_ESM.docx]

Supplemental Digital Content 1


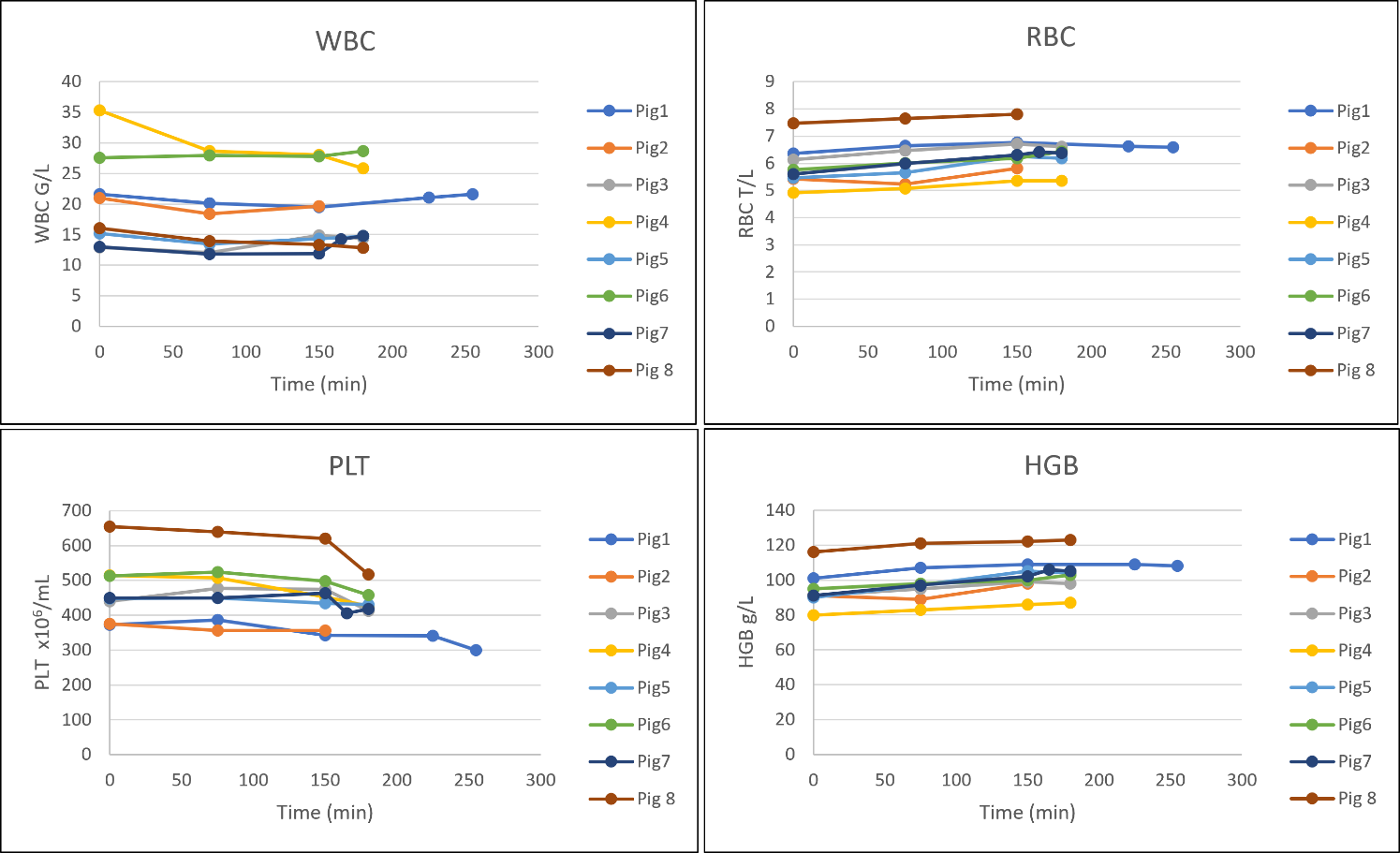


**Supplemental Figure 1. White blood cell (WBC), red blood cell (RBC), hemoglobin (HGB) and platelet count (PLT) during experiment time in pigs 1-8.**

Blood cell counts and hemoglobin were followed with pigs (n=8) at the time points indicated by solid symbols. Baseline sample was collected immediately before the first APAC (i.v.) dose at 0 min time point. In pig 1, APAC was administered at escalating doses of 0.15 mg/kg at 0 min, 0.25 mg/kg at 75 min, 0.5 mg/kg at 150 min and 0.75 mg/kg at 225 min time points. In pigs 2-6, APAC was administered at escalating doses of 0.25 mg/kg at 0 min, 0.5 mg/kg at 75 min and 0.75 mg/kg at 150 min time points. In pigs 7 and 8, APAC was administered at escalating doses of 0.5 mg/kg at 0 min, 0.75 mg/kg at 75 min and 1.25 mg/kg (pig 7) or 1.5 mg/kg (pig 8) at 150 min. Protamine sulfate (140 IU/kg) was administered to all animals at 15 min after the highest APAC dose (pig 1 at 240 min, and pigs 2-8 at 165 min).
