# Supplementary material for: Escalating doses of intravenous APAC demonstrate antithrombotic effect in pigs
Source: Thromb J. 2025 Jun 4;23:57. doi: 10.1186/s12959-025-00742-8 (PMC12135276; doi:10.1186/s12959-025-00742-8)
Supplement: Supplementary file 2 — Supplemental Table S1 (PDF): Effect of escalating doses of intravenous APAC on thrombin generation in plasma and its reversal by protamine sulfate in pigs 2-6. [file 12959_2025_742_MOESM2_ESM.docx]

Supplemental Digital Content 2

**Supplemental Table 1. Effect of escalating doses of intravenous APAC on thrombin generation in plasma and its reversal by protamine sulfate in pigs 2-6.**

| n=5 | Time (min) |  | Lag time (min) | ETP (nM*min) | Peak (nM) | ttPeak (min) |
| --- | --- | --- | --- | --- | --- | --- |
| Baseline | 0 | Mean | 2.0 | 373 | 139 | 3.6 |
|  |  | SD | 0.4 | 119 | 43 | 0.4 |
| 0.25 mg/kg | 15 | Mean | 2.3 | 362 | 125 | 3.9 |
|  |  | SD | 0.5 | 105 | 28 | 0.5 |
|  | 75 | Mean | 2.1 | 372 | 138 | 3.7 |
|  |  | SD | 0.5 | 99 | 31 | 0.6 |
| 0.5 mg/kg | 15 |  | 3.5 - 8.8* | 51 – 319* | 3 – 29* | 9.8 – 16* |
|  | 30 | Mean | 2.1 | 416 | 143 | 3.8 |
|  |  | SD | 0.5 | 140 | 39 | 0.7 |
|  | 75 | Mean | 2.0 | 434 | 153 | 3.5 |
|  |  | SD | 0.3 | 168 | 38 | 0.5 |
| 0.75 mg/kg | 15 | abolished TG | | | | |
| Protamine sulfate | 30 | Mean | 2.0 | 462 | 161 | 3.5 |
|  |  | SD | 0.3 | 196 | 43 | 0.5 |

Thrombin generation (TG) test was conducted utilizing the Thrombinoscope CAT assay (Calibrated Automated Thrombogram, Maastricht, The Netherlands), according to the manufacturer's instructions (Diagnostica Stago, Asnières, France). In brief, PPP-Reagent^TM^, containing 5 pM recombinant tissue factor and 4 µM phospholipids was used. Baseline sample was collected immediately before the first APAC (i.v.) dose. APAC was administered at escalating doses of 0.25 mg/kg at 0 min, 0.5 mg/kg at 75 min and 0.75 mg/kg at 150 min time points. Protamine sulfate (140 IU/kg) was administered at 15 min after APAC dose of 0.75 mg/kg and TG detected during the next 15 min. * In 2 pigs TG was completely abolished at 0.5 mg/kg dose level after 15 min; the detectable TG range in 3/5 pigs are shown.

Time = time passed after the indicated APAC administration, Lag time = the point at which the signal deviates by more than 2 standard deviations from the baseline, ETP = endogenous thrombin potential (area under the curve), Peak = maximal thrombin generation, ttPeak= time to peak.
