# Supplementary material for: Escalating doses of intravenous APAC demonstrate antithrombotic effect in pigs
Source: Thromb J. 2025 Jun 4;23:57. doi: 10.1186/s12959-025-00742-8 (PMC12135276; doi:10.1186/s12959-025-00742-8)
Supplement: Supplementary file 3 — Supplemental Figure S2 (PDF): Effect of escalating doses of intravenous APAC on activated partial thromboplastin time (APTT), thrombin time (TT) and prothrombin time (PT) in plasma. [file 12959_2025_742_MOESM3_ESM.docx]

Supplemental Digital Content 3


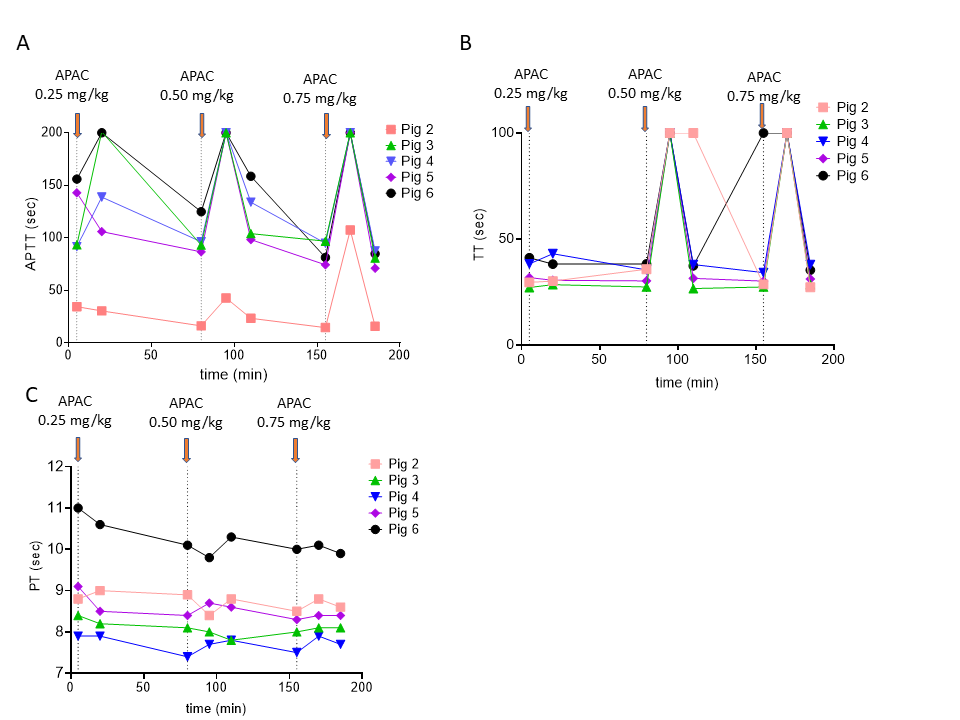


**Supplemental Figure 2.** **Effect of escalating doses of intravenous APAC on activated partial thromboplastin time (APTT), thrombin time (TT) and prothrombin time (PT) in plasma.**

APTT (A), TT (B) and PT (C) were followed in plasma after administering escalating doses of APAC (i.v.) (0.25, 0.5 and 0.75 mg/kg) (Pigs 2-6; n=5). Baseline sample was collected immediately before the first APAC dose. The timepoints of APAC dosing are illustrated by orange arrows. Blood collection times and respective results are presented by solid symbols. Protamine sulfate (140 IU/kg) was administered to all animals at 15 min after the highest APAC dose, before the last blood sample was collected. Maximal APTT and TT detection times were 200 s and 100 s, respectively.
